# Large Language Models for Depression Detection: A Review with Prospects of Incomplete Multimodality

**DOI:** 10.3390/brainsci16060593

**Published:** 2026-05-30

**Authors:** Anqi Dai, Weipeng Shi, Xiaogang Gu, Lingqin Huang

**Affiliations:** School of Electrical Engineering and Automation, Jiangsu Normal University, Xuzhou 221116, China; 2020241942@jsnu.edu.cn (A.D.); shiweipeng@jsnu.edu.cn (W.S.); guxg@jsnu.edu.cn (X.G.)

**Keywords:** depression detection, large language models, affective computing, incomplete multimodal learning, deep learning

## Abstract

**Highlights:**

**What are the main findings?**

- Depression represents a growing global mental health burden, and considerable progress has been achieved in depression recognition using unimodal, multimodal analyses and large language model (LLM)–based methods.
- However, research addressing incomplete multimodal data in real-world clinical settings remains limited, which represents a key gap in current depression-recognition methodologies.

**What are the implications of the main findings?**

- Future research should address incomplete multimodal learning strategies to handle missing or noisy modalities in real-world clinical scenarios.
- LLM-driven adaptive and knowledge-aware frameworks will be essential for building robust, clinically reliable and interpretable depression-recognition systems.

**Abstract:**

Depression has emerged as one of the leading contributors to the global burden of mental disorders, ranking among the top causes of disability worldwide. With its steadily increasing prevalence, accurate and efficient depression detection has become an urgent yet challenging task. In this paper, we provide a comprehensive review of depression-recognition research from multiple perspectives. Specifically, we first outline the epidemiological status of depression and summarize the commonly used depression assessment scales and public datasets. Then, we structurally review the research progress of depression recognition from the perspectives of unimodal analysis and multimodal fusion, with a particular focus on large language model (LLM)-based methods and their potential in addressing challenges arising from incomplete multimodal data. Furthermore, we identify a critical gap in depression recognition under incomplete modality conditions, which are common in real-world clinical scenarios, and outline future directions toward LLM-driven and clinically applicable solutions. Finally, the clinical translation, ethical considerations, and human-centered deployment of LLM-based depression-recognition systems under real-world healthcare constraints are discussed.

## 1. Introduction

Depression, a prevalent mental disorder characterized by persistent low mood, diminished interest in daily activities [1], and interpersonal perception and behavioral distortions linked to negative social expectations, severely impairs individuals’ daily functioning and elevates the risk of morbidity and mortality from multiple somatic diseases, including cardiovascular, metabolic, autoimmune, inflammatory, and neurological disorders [2]. Epidemiologically, approximately 5% of the global population experiences a depressive episode within any 12-month period; as reported by the World Health Organization, depression became the second leading cause of global disease burden and disability by 2020, after ischemic heart disease, and is projected to become the leading contributor by 2030 [3], with the 2025 data showing over 350 million people worldwide living with depression, among whom about 27% are in China. Despite its high prevalence and severe health impacts, depression detection and management remain formidable challenges in modern community healthcare due to the subjective nature of its symptoms: around 33% of all depressed patients fail to be diagnosed clinically, and less than 40% of the total patient population receive appropriate treatment [4], and a World Psychiatric Association survey indicates nearly half of Chinese respondents are unwilling to actively seek medical care after diagnosis [5]. This reluctance may be partly associated with traditional perceptions and social stigma surrounding mental illness, which can discourage some individuals from seeking psychological counseling or psychiatric assistance at an early stage. Against this backdrop, online mental health service models have gradually emerged as a feasible supplementary approach. Automatic depression screening systems [6,7,8], as an important component of such services, can provide individuals with an anonymous and low-threshold preliminary assessment of their emotional condition and may help promote earlier professional intervention when necessary. Although automated self-screening tools may still involve inherent biases associated with self-reported data, they can partially alleviate current challenges related to limited mental health resources and insufficient early screening accessibility.

Recently, automated depression recognition has garnered growing interest across medicine, psychology, and computer science. Given distinct multimodal differences in facial, postural, speech, and physiological features between depressed and healthy individuals, researchers have extensively leveraged such data for depression severity prediction [9,10,11]. These cutting edge technologies have enabled precision medicine, which emphasizes personalized patient specific therapies over generalized population based treatment strategies [12]. In recent years, the paradigm of precision medicine has further catalyzed the development of personalized mental health care, a customized intervention model that is widely recognized as precision psychiatry. Currently, research integrating Artificial intelligence (AI), particularly Large language model (LLM), and big data analytics into mental health interventions is rapidly expanding. This escalating research momentum is corroborated by Brunn et al. [13], who performed a bibliometric analysis of the PubMed database and reported a 250% surge in the number of publications pertaining to the intersection of AI and psychiatry over the period from 2015 to 2019 [14].

**Figure 1:**
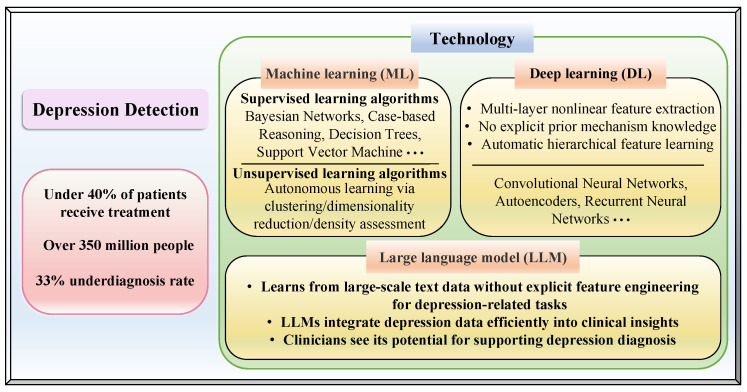
Current status of depression and depression-recognition technologies.

With the rapid evolution of AI, LLMs have emerged as a promising paradigm in mental health, particularly in depression recognition [15]. As shown in Figure 1, their ability to process complex and largely unstructured data offers new opportunities for advancing psychiatric analysis. LLMs are fundamentally rooted in the broader evolution of machine learning (ML) and deep learning (DL), which have been applied to support depression-recognition research [16,17,18].

As an important branch of AI, ML provides a practical technical framework covering data analysis, pattern mining, and model construction; as an autonomous learning approach based on historical datasets [19], its core principle involves inputting large scale data, processing it via algorithms, training models, and achieving predictive functionality-particularly valuable in depression diagnosis and prediction [20]. ML is categorized into supervised and unsupervised learning: supervised learning relies on labeled data to establish input-output mapping (e.g., Support Vector Machine (SVM) [21], Logistic Regression (LR) [22], Decision Trees (DTs) [23]), while unsupervised learning, which requires no data labeling, has higher computational costs but enables autonomous learning through clustering, dimensionality reduction, and density assessment (e.g., K-means, Principal Component Analysis (PCA)) [20]; both types have provided fundamental technical support for AI-aided depression recognition [24,25,26,27,28,29]. As a prominent branch of ML, DL constructs layer-structured artificial neural networks capable of autonomous learning and intelligent decision-making, extracting abstract data features through multi-layer nonlinear processing to build high-precision models from large-scale datasets without prior knowledge of internal data mechanisms; mainstream algorithms like Convolutional Neural Networks (CNNs) [30] have shown effectiveness in depression detection, laying a critical technical foundation for the subsequent rise of LLMs in mental health research [31,32].

Building upon advances in ML and DL, LLMs have emerged as a new paradigm in AI. As a core technical carrier, LLMs are capable not only of processing multimodal data related to depression—including clinical scales, patient narratives, social media content, and physiological signals [33,34,35,36]—but also of capturing subtle linguistic and emotional cues and transforming them into clinically valuable insights [37,38]. As such, LLMs demonstrate clear potential in addressing the challenges posed by incomplete multimodal data and in advancing precision psychiatry and depression detection. This potential is supported by growing clinical consensus: Doraiswamy et al. [39] reported that the majority of psychiatrists believe LLMs will significantly enhance the efficiency and accuracy of depression diagnosis.

However, in real-world clinical settings, multimodal data are often incomplete due to various factors, such as patients’ unwillingness to record videos, poor audio quality, privacy concerns, and camera equipment failures. These limitations, together with the inherent heterogeneity of multimodal data, collectively constrain the practical application of LLMs in depression recognition. Against this technological and clinical background, this review summarizes the research progress on LLM-assisted depression recognition, analyzes the prospects of depression recognition under conditions of incomplete multimodal data, clarifies that research specifically addressing incomplete multimodality in depression detection remains limited, and then outlines the current state, challenges, and development trends in this field.

This study is a comprehensive narrative review focusing on depression recognition using LLMs, multimodal learning methods, and incomplete multimodal analysis. Relevant literature was retrieved from major academic databases, including Web of Science, PubMed, IEEE Xplore, and Scopus, with supplementary searches conducted through Google Scholar. Most selected studies were published within the past decade to reflect recent advances in AI and depression detection, while several seminal earlier works related to depression assessment scales and benchmark datasets were also retained. The collected literature was manually screened based on its relevance to depression recognition, multimodal fusion, incomplete modality learning, and LLM-based mental health analysis. On this basis, the selected studies were summarized according to different research directions, with discussions on current progress, mainstream approaches, existing limitations, and future development trends in this field.

## 2. Depression Assessment Scale

The process of annotation of depression datasets is a key and multi-step task, the quality of which directly influences further studies. A speech-based depression database that has been annotated in a comprehensive fashion usually comprises three parts: transcription, analysis, and annotation. The process of transcribing audio and linguistic data into written expression is known as transcription; the subsequent labeling of acoustic data (e.g., prosodic data, speaking rate, volume, and intonation change) in accordance with the text that has been transcribed is called analysis; the act of attributing the degree of depression to a single sentence is called annotation [40]. The AVEC 2013 dataset [41] was annotated using the Beck Depression Inventory-II (BDI-II) [42] from two dimensions: Arousal and Valence. For the DAIC-WOZ dataset [43], the ELAN tool was employed for the transcription phase, where the interviewee’s psychological states, dialogue content, and non-verbal behaviors were analyzed and labeled. Regarding the MODMA dataset [44], all recordings were manually segmented and annotated in accordance with the Diagnostic and Statistical Manual of Mental Disorders (DSM-IV) [45]. Table 1 presents the score ranges of different questionnaires and their corresponding depression severity levels.

## 3. Depression Dataset

At present, a large number of depression-related datasets have been developed and applied, including commonly used ones such as AVEC2013 [41], AVEC2014 [51], CHI-MEI [52], and DAIC-Woz [43]. These datasets provide essential data support for the application of LLMs in depression recognition, especially for research on incomplete multimodality. This paper will focus on introducing several widely used depression datasets, as shown in Table 2, and systematically list and compare the selected datasets from six core dimensions: subjects, annotation, language, modality, public/private (open access permission), and time.

The datasets currently available in depression recognition have created a multilingual study framework that encompasses German, English, and Chinese, and a well-developed hierarchical framework in terms of sample size, modal type, and annotation specifications. AVEC2013 [41], and AVEC2014 [51] are classic benchmark datasets on German, which offer annotations of 292 subjects with BDI-II [42] on each dataset. They address audio and visual modalities, and use standardized datasets partitioning schemes, which form a basis for the recognition of audiovisual multimodal depression. AVEC2017 [53] and AVEC2019 [54] further expanded data collection methods and feature dimensions, collecting data through virtual interviews and adding DL features to adapt to the training requirements of different models. English datasets have formed a multi-level annotation system ranging from basic scales to clinical diagnoses. DAIC-Woz [43], with its full-modal (audio, visual, text) data and PHQ-9 annotations, has become a widely used resource for multimodal fusion research. The Pittsburgh [55] dataset screens subjects based on the DSM-IV [45] diagnostic criteria, while the BD [56] dataset conducts diagnoses in accordance with the DSM-V and incorporates MADRS [50]/YMRS scales to achieve refined symptom annotation; both are public datasets. DEPAC [57] assesses the severity of depression using the PHQ-9 [47] and anxiety severity using the GAD-7, and its large-scale single-modal audio data provides support for the training of lightweight models. Blackdog [58] focuses on clinical dialogue data in the audio modality based on the DSM-IV [45] criteria. Chinese datasets have risen notably in recent years. MODMA [44] integrates audio and electroencephalogram (EEG) signals to expand the modal boundary. MMDA [59], CMDC [60], and EATD [61] are based on semi-structured/structured interviews, covering full or core modalities, and meet the needs of depression screening in the Chinese context. Moreover, most of these datasets are publicly available, lowering the threshold for research.

**Table 2:** Overview of Depression Datasets.

Dataset	Subjects	Annotation	Language	Modality	Public/Private	Time
AVEC2013 [41]	292	BDI-II	German	A + V	Public	2013
AVEC2014 [51]	292	BDI-II	German	A + V	Public	2014
AVEC2017 [53]	-	PHQ-8	English	-	Public	2017
AVEC2019 [54]	-	PHQ-8	English	-	Public	2019
DAIC-WoZ [43]	110	PHQ-9	English	A + V + T	Public	2014
Rochester [62]	27	Manual annotation	-	V	Private	2015
CHI-MEI [52]	53	DSSS, HAMD	-	V	Private	2016
Pittsburgh [55]	57	DSM-IV, HAMD > 15	English	A + V	Public	2018
BD [56]	46	DSM-V	-	A + V	Public	2018
MODMA [44]	163	PHQ-9	Chinese	A + EEG	Public	2020
Blackdog [58]	80	complete the task	English	A + V	Private	2009
DEPAC [57]	571	complete the task	English	A	Public	2023
MMDA [59]	524	Interview and complete the task	Chinese	A + V + T	Public	2022
CMDC [60]	78	Semi-structured interview	Chinese	A + V + T	Public	2023
EATD [61]	162	Semi-structured interview	Chinese	A + T	Public	2022

These datasets, featuring standardized clinical scales and diagnostic labels, diverse modal combinations, and cross-lingual sample coverage, provide a high-quality foundation for depression recognition using LLMs, opening research directions such as textual semantic analysis and multimodal feature fusion. However, despite the growing number of public depression datasets, most remain limited in sample size, demographic diversity, and clinical standardization, and are often collected under controlled laboratory conditions that differ substantially from real-world clinical environments—challenges that may affect the generalization and practical deployment of current models. Moreover, existing studies predominantly rely on complete multimodal data, yet incomplete modalities are common in practice, posing a new challenge for depression detection technologies and highlighting the need to prioritize research on handling missing modalities as a future direction.

## 4. Depression Detection

Depression can be identified and judged based on diverse information, including facial expressions, vocal features, linguistic expressions, and physiological signals, and numerous relevant studies have been conducted worldwide in this field. As illustrated in Table 3, these studies can be primarily categorized into two detection paradigms: unimodal and multimodal depression detection. Both paradigms are rooted in the multimodal datasets elaborated in Section 3, yet they have inherent limitations that give rise to an urgent demand for advanced LLM-driven solutions—one that will be further explored in depth via LLMs in Section 5.

### 4.1. Unimodal Depression Detection

In the field of automated depression detection, unimodal technologies represent a modality-centric paradigm in depression detection, which focuses on extracting depression-related features from a single data source. This is largely due to the high-quality unimodal data from the datasets in Section 3, which endow unimodal research with convenient data collection, basic model architecture, and ease of implementation. These technologies center on a single modality of biological or behavioral data. Through mining depression-related pathological features, they achieve objective detection and quantitative evaluation, while also providing a basis for LLM-based feature extraction and fusion. Currently, unimodal research focuses on four major modalities: vision, audio, text, and EEG. Representative technical systems have been established, supporting objective diagnosis and paving the way for the LLM-driven transition to multimodal depression detection.

The fundamental weakness of unimodal approaches is that they do not reflect the multidimensional nature of depression, which is in line with the data characteristics (single-modal to multimodal development) presented in Section 3. This weakness prompts the adoption of multimodal solutions and encourages the use of LLMs since the semantic comprehension and the ability to integrate features can be used to address unimodal weaknesses and improve depression detection technology.

#### 4.1.1. Visual Modality

One of the most researched directions of unimodal depression recognition is the visual modality. This technology uses mostly visual data like facial expressions and facial movements, and images of scenes on a daily basis to mine and extract discriminative extraneous behavioral characteristics of depressed patients. The initial research was largely based on the analysis of facial expressions in a state. As an example, Fu et al. [67] asked the participants to take a voluntary imitation task with seven types of expressions. The researchers assessed the level of their imitation and discovered that patients with depression had multidimensional impairments in terms of facial expression imitation, which might be an essential source of reference to detect the disease. Nevertheless, it was a study that used fixed stimulus materials, therefore, lacking the weakness of low ecological validity. As the DL methods have progressed, more studies have been performed on the basis of dynamic facial videos and natural scene images. PRA-Net model suggested by Liu et al. [68] is the first to use a part-relation attention network: it partitions feature maps to extract semantically rich local features, and it uses a combination of self-attention and relational attention to optimize the correlation weights between local and global features. This methodology overcomes the limitation of the lack of representation capability in traditional methods and has the highest state-of-the-art performance in end-to-end methods on the AVEC2013 and AVEC2014 datasets. Likewise, the model of the Spatial-Temporal Attention Depression-Recognition Network (STA-DRN) developed by Pan et al. [69] incorporates both the global and local spatiotemporal characteristics of facial videos through a spatiotemporal attention mechanism. It takes an attention vector fusion approach to incorporate spatiotemporal domain information, which greatly promotes the ability of the model to capture dynamic features of faces that relate to depression. Song et al. [70] suggested a generalized loss function relaxation strategy to solve the problem of noise interference in video data collected under real-world conditions. It is able to automatically adjust training parameters, counter the adverse effects of noisy data and labels on model training, and efficiently improve model performance in 25 different artificially simulated noise conditions, which inherently enhances the robustness of models based on visual modality to recognize depression. Moreover, the MoodCapture approach suggested by Nepal et al. [84] overcomes the shortcomings of laboratory-obtained data. It gathers in-the-wild facial images in everyday life through smartphone front-facing cameras, trains a random forest model on facial key points, and experiences both depression classification and PHQ-8 scale score prediction accuracy. In the meantime, this research paper makes a thorough study of the issue of privacy, which is useful in designing mental health assessment instruments in real life. Interestingly, these high-tech visual-modality technologies and feature extraction techniques form a good basis to be incorporated into the LLM in the depression detection process, which offers viable visual feature support for research on depression detection using LLMs.

#### 4.1.2. Audio Modality

The audio modality centers on speech signals, leveraging the specificities of patients with depression in terms of vocal frequency, speech rate, and emotional expression to construct automated recognition models. Feature extraction from speech signals and model optimization constitute the research priorities in this field. Arnab Kumar Das et al. [63] proposed a method that fuses Mel-frequency cepstral coefficients (MFCCs) and spectrogram features generated by CNNs. By optimizing a CNN architecture with residual blocks and kernel initializers for feature extraction and classification, this method effectively addresses the diagnostic challenges in depression identification caused by patients’ social desirability bias and insufficient cognitive awareness of their conditions. Huang et al. [64] adopted wav2vec 2.0 as the feature extractor and constructed a classification model by combining it with a lightweight fine-tuning network. Eliminating the need for complex feature preprocessing operations, this approach achieved high-precision binary and multi-classification recognition on the DAIC-Woz dataset, which effectively addressed the problem of insufficient dataset scale. To further enhance the anti-interference capability and recognition accuracy of the model, Pan et al. [85] proposed the multi-feature deep-supervised voiceprint adversarial network (MFDS-VAN). This network fuses long-term and short-term acoustic features with raw audio waveforms via an encoding network, mitigates individual voiceprint interference through the voiceprint adversarial module, and optimizes the network architecture by means of a deep-supervised regression algorithm, thus achieving outstanding performance on multiple AVEC datasets. Starting from the problem of data sparsity, Wu et al. [65] proposed a method that combines self-supervised pre-trained foundation models with knowledge transfer. By analyzing the representations of different layers of foundation models, transferring knowledge from speech recognition and emotion recognition tasks, and fusing representations from multiple foundation models, this method achieved favorable recognition performance on the DAIC-Woz dataset, providing new insights for the training optimization of audio-modality-based models. The study conducted by Menne et al. [86] further verified the feasibility of speech features as biological markers for depression. The ML model he proposed discriminates between patients with major depressive disorder and healthy controls based on multidimensional speech features, achieving performance comparable to that of traditional scale-based assessments and thereby providing a potential tool for clinical diagnosis and disease monitoring. As a key component of depression detection systems, mature audio feature extraction and model optimization technologies can be effectively integrated with LLMs’ semantic understanding capabilities, which is crucial for solving the problem of incomplete modality depression detection where audio data may be missing or incomplete.

#### 4.1.3. Text Modality

The text modality is mainly based on mining depressive hints implied in users’ linguistic expressions using social media text data, and its advantages are non-invasive and easily available data. The key issue in the research community is how to extract complete and effective linguistic features. However, the existing models have two main problems: single-dimensional features and limited generalization ability. Lyu et al. [66] extracted multidimensional lexical features based on Chinese microblog text data with the aid of multiple tools, including the Chinese version of the Linguistic Inquiry and Word Count (LIWC) dictionary, suicide-related dictionaries, and culture-psychology-related dictionaries, and further constructed prediction models accordingly. Among these models, the linear regression model demonstrated optimal performance, which verified the significant value of culture-psychology and suicide-related lexical features for depression prediction, thus providing a more comprehensive reference for depression recognition using text-only social media data. Research on the text modality has paved a feasible way for large-scale population screening of depressive risks. However, current studies still rely on platform-specific data, and the cross-platform generalization capability of the models awaits further verification. Text data are highly compatible with LLMs, and the linguistic feature extraction methods developed in text-modality depression recognition can be effectively integrated with LLMs to enhance their ability to identify depressive tendencies. LLMs’ powerful semantic understanding capability can compensate for the limitations of a single text modality, providing a more robust technical support for text-based depression detection.

#### 4.1.4. EEG Modality

The EEG modality relies on the physiological specificity of EEG signals to directly reflect the pathological changes associated with depression in cerebral neural activities, thus demonstrating high objectivity and accuracy. Research priorities in this field focus on feature fusion, channel optimization, and the enhancement of model generalization capability. To address the problems of channel redundancy, high computational complexity, and the neglect of the complementarity of spatiotemporal and spectral features in existing models for EEG data analysis, Xi et al. [71] proposed a detection method that fuses the spatiotemporal and spectral features of EEG signals. Specifically, this method optimizes the extraction of spatial-spectral features via frequency-domain weighted channel selection and designs a Multi-Scale Spatiotemporal Convolutional Attention Network (MSTAN) to capture spatiotemporal features. In contrast, the EEG-based Depression Transformer (EDT) model proposed by Ying et al. [72] focuses on addressing the insufficient exploitation of spectral domain information in EEG data. It extracts spectral and spatial domain features via an information extraction module, perceives feature dependencies and fuses the features with the aid of an attention module, and extracts temporal domain features simultaneously. Validated by ten-fold cross-validation, the model achieves higher accuracy than both baseline models and its own variants, demonstrating promising potential as a feasible solution for EEG-based depression recognition. To address the insufficient generalization capability of existing DL models for EEG-based depression recognition, which is caused by the failure to fully consider the differences in the importance of different EEG channels, intra-channel features, and cross-subject data distribution differences, Zhang et al. [73] proposed the SSPA-GCN model. This model uses a two-dimensional attention matrix to focus on key channels and features and integrates the Subject Sub-Partitioning (SSP) domain generalization module to group subjects with similar data distributions. As a result, it strengthens the model’s ability for cross-subject recognition. Given the highly objective nature of this physiological modality, the EEG-based feature extraction technique offers clinically applicable physiological backing for LLM-based depression assessment. In forthcoming studies on incomplete multimodal depression detection, integrating EEG features with other available modalities can address issues with absent data, enhancing the accuracy and reliability of depression detection.

Combined with relevant techniques, unimodal mood disorder detection has achieved remarkable advancements in four key areas. Numerous technical approaches and model designs have been proposed, providing a strong basis for the objective detection of mood disorders. However, current unimodal studies have several common drawbacks. Data collected from a single modality fail to capture the full range of complex, pathological, and behavioral features of mood disorders. For example, the visual paradigm does not take into account physiological responses, and the EEG modality does not include behavioral components. Second, the lack of generalization of the models is also a drawback. Most studies are based on data collected in a laboratory setting, and their performance in real-world situations involving diverse datasets is still unknown. Third, the recognition performance of some models is not notable; they have minor deficiencies and are still affected by problems such as excessive complexity and long training times. All of these combined inadequacies point to the fact that unimodal systems lack the necessary level of precision and reliability needed for clinical and large-scale screening. Hence, the integration of multiple approaches is recognized as a promising avenue to pursue in the field of clinical mood disorder detection. Future unimodal research should concentrate on the lightweight optimization of current models, the enhancement of adaptability to real-world situations, and the seamless integration of multimodal data to establish the groundwork for developing a more sophisticated and precise system for recognizing depression. More importantly, these limitations are exacerbated in real-world clinical scenarios with incomplete multimodal data, which further motivates the application of LLMs and their multimodal variants to address such challenges.

### 4.2. Multimodal Depression Detection

Although unimodal depression detection technology has achieved significant progress in fields such as vision, audio, text, and EEG, single-modal difficult to comprehensively characterize the complex pathological mechanisms, physiological characteristics, and behavioral manifestations of depression. It generally has limitations such as one-sided representation and restricted generalization ability, making it difficult to meet the requirements of high precision and high robustness for clinical diagnosis. Against this background, multimodal fusion technology has become a research hotspot in the field of depression recognition due to its advantages of complementarity and comprehensiveness. By integrating two or more heterogeneous data types, multimodal depression recognition explores the synergistic correlations between modalities, which can effectively make up for the information loss of unimodal data and improve the recognition performance and clinical applicability of the model.

This multimodal fusion paradigm aligns with the multi-source, heterogeneous datasets described in Section 3, where combining clinical, behavioral, and physiological data is standard, yet traditional fusion methods still face challenges such as modality heterogeneity and incomplete data. These limitations, which hinder the further improvement of multimodal depression detection performance, will be addressed by multimodal LLMs (MLLMs), which will be the focus of the next chapter.

The core rationale of current research on multimodal depression detection lies in data complementarity and fusion synergy. According to the differences in data modality combinations, the related methods can be categorized into two paradigms: physiological signal and behavioral modality fusion and multi-behavioral modality fusion. All these studies focus on exploring the core issue of how to design effective fusion strategies to capture the synergistic value among different modalities.

#### 4.2.1. Physiological Signal and Behavioral Modality Fusion

The fusion of physiological signals and behavioral modalities aims to achieve the synergistic representation of objective physiological indicators and external behavioral manifestations, so as to overcome the limitations of unimodal diagnosis and improve the reliability and comprehensiveness of depression recognition. As a basic fusion paradigm, single-physiology and single-behavior fusion typically employs combinations such as EEG and facial features, fNIRS and audiovisual data. To address the deficiencies of unimodal systems, Hamid et al. [75] designed a hybrid DL model that fuses EEG and facial biometrics and implemented feature fusion via BiLSTM. Experiments verified that the diagnostic accuracy of the proposed model was significantly superior to that of unimodal models, confirming the complementary value of physiological and behavioral features.

On this basis, single-physiology and multi-behavior modal fusion further expand the information dimension. Wang et al. [74] proposed the MFCAF hybrid model, which synchronously collects video, audio, and fNIRS data under consistent stimulation conditions. Features of the three modalities are extracted via multi-scale CNN-GRU, ViT, and multi-channel CNN, respectively, and the dependencies between modalities are captured through a Transformer-based cross-attention module. The model outperformed baseline methods on a self-constructed dataset and effectively identified subclinical and atypical depressed populations, providing a new approach for clinical auxiliary diagnosis of depression. To pursue comprehensive data coverage and strong model generalization, multi-physiology and multi-behavior full-modal fusion integrates four or more types of multimodal data for a more thorough representation of depression. Kumar et al. [76] proposed the multimodal interpretable depression detection (MultiDepNet) system, which innovatively fuses visual, physiological, audio, and textual modalities. For each modality, the use of MTCNN and TS-CAN is proprietary, and for strategic fusion, different combinations of CNN-RNN and Transformer, among others, are used. The system demonstrated remarkable results on four benchmark datasets.

These paradigms boost the clinical translation of multimodal depression recognition as they objectively and comprehensively enhance the diagnosis of depression. From the complementary validation of depression diagnosis through single-physiology and single-behavior fusion, fine-grained feature representation with single-physiology and multi-behavior fusion, and the overall integration with multi-physiology and multi-behavior fusion.

In practical application, these traditional physiological-behavioral fusion paradigms still face prominent challenges in handling incomplete multimodal data in clinical settings due to patient non-cooperation and data collection obstacles. This has driven the exploration of LLMs and MLLMs for depression detection and multimodal depression detection, with a detailed investigation presented in the next chapter: with strong semantic modeling and cross-modal reasoning capabilities, LLMs and MLLMs act as powerful supplements to traditional fusion models.

#### 4.2.2. Multi-Behavioral Modality Fusion

“Multi-behavioral modality” fusion focuses on various external behavioral data, emphasizing the exploration of correlations between modalities such as face, body, voice, and text. Common combinations include “face + body”, “audio + text”, “face + audio + text”, etc. In the direction of “face + body” fusion, both Xingyun Li and Yang Liu focused on the problem that existing studies ignore body expressions. They collected data through emotional stimulation experiments and confirmed that there are significant differences and synergistic relationships in facial and body expressions between depressed patients and healthy people. Li et al. [77] proposed the two-stream feature fusion model (TSFFM), whose core FE module enhances local features via an embedded attention mechanism, extracts temporal features, and fuses effective spatiotemporal information. The model achieved an accuracy and F1-score of 0.896 on the self-built emotion stimulation dataset. Liu et al. [78] employed the pre-trained ResNet-50 network and OpenFace toolkit for feature extraction and sequence analysis and constructed a DL model for depression recognition. Experiments revealed that depressed patients exhibited significant activations in negative facial action units (e.g., AU04, AU07). The decision-level fusion model based on facial and body features achieved a maximum accuracy of 0.904 and an F1-score of 0.901, validating the effectiveness of the multimodal method for the preliminary screening of depression.

In the direction of “audio + text” fusion, aiming at the problem that patients are likely to provide false answers when filling out questionnaires, Iyortsuun et al. [79] proposed an additive cross-modal attention network model with BiLSTM as the backbone network, realizing the fusion of audio and text features under the premise of eliminating the interference of interview questions. It performed excellently on the DAIC-Woz and EATD-Corpus datasets, providing a feasible solution for depression detection without preset questions. In the direction of “face + audio + text” multi-behavioral modality fusion, Zhang et al. [80] proposed an ensemble voting fusion model, which processes text features through BiLSTM, audio features through PCA + SVM, and video features through XGBoost, and finally fuses and outputs the results. It achieved an excellent weighted F1-score of 0.85 on the DAIC-WOZ dataset, breaking through the representational limitations of single behavioral modalities. Focusing on the accuracy of PHQ-8 score prediction, Li et al.’s [87] FPT-Former model fuses expert knowledge-driven descriptors of video and two audio modalities and realizes feature fusion and regression through the Transformer encoder. Ablation studies confirmed that three-modal fusion, as opposed to either single or double-modal fusion, provides an accurate means to gauge the severity of depression. Maximizing the dimensions of data in such studies furthers the complex depiction of depression and increases the development of multimodal recognition to a level of greater accuracy and clinical utility.

Regarding the optimization of fusion techniques, current studies have progressed from conventional early feature fusion followed by late decision-level fusion to more complex forms such as hybrid, knowledge-based, and attention-based fusions. Zhang et al. [81] discussed the gaps in multimodal depression detection, including the insufficient amount of training data, modality selection restrictions because of privacy and ethical considerations, and the necessity to integrate contextual information and long-term dependencies. They presented a hybrid fusion model that combines facial and speech bimodal data. The model utilizes the early intra-modal fusion + late inter-modal fusion strategy, uses CNN and BiLSTM to address both frame-level and long-term dependencies, and applies the ADF attention module to equilibrate the influence of each modality for improved performance over classical single fusion approaches; to mitigate the higher computational burden of the Transformer’s self-attention mechanism, Liu et al. [82] designed the attention-free Multimodal Fusion Mamba (MFMamba) framework, which employs a state space model to efficiently extract and integrate audiovisual modalities and achieved state-of-the-art results on the AVEC benchmark; Yang et al. [83] introduced the Hierarchical Knowledge Enhanced Prompt Fusion (HKEPF) model where the fusion of multimodal components was further boosted by the integration of specialized knowledge from the domains of psychology and psychiatry, which resulted in fine-grained multimodal fusion, improved feature correlation to depression, and greater model explainability. Continuous evolution of fusion methods has become the main factor leading to advancements in performance for multimodal models.

Despite significant advances in multimodal depression detection, existing approaches still face substantial challenges in handling incomplete multimodal data in real-world clinical settings. Different modalities are often collected asynchronously and may contain missing, noisy, or low-quality signals due to poor patient compliance, limitations in data collection, privacy concerns, and variations in clinical environments. Therefore, improving the robustness of depression detection systems under incomplete multimodal conditions remains a critical research direction for practical clinical applications. Meanwhile, the intelligence and adaptability of current detection systems are yet to be fully improved to cater to the dynamic and complex nature of real-world clinical scenarios. In fact, through the integration of heterogeneous data and optimization of fusion strategies, multimodal depression recognition has effectively made up for the shortcomings of unimodal research, achieved significant breakthroughs in recognition accuracy, generalization ability, and clinical applicability, and provided multiple technical paths for the accurate diagnosis of depression. However, several urgent challenges persist: (1) At the data level, constrained by privacy and ethical concerns, most research datasets are characterized by small sample sizes and limited population diversity. Meanwhile, the high acquisition cost and difficult annotation of multimodal data further limit the generalization ability of models; (2) At the fusion level, some models suffer from issues including unbalanced modal contributions, such as inadequate contribution from the audio modality, high computational costs induced by complex fusion mechanisms, and limited interpretability; (3) At the clinical transformation level, most existing models are verified based on benchmark datasets, and their adaptability and practicality in real clinical scenarios need to be further verified.

Against this background, the advancement of multimodal depression recognition relies heavily on the deep integration of advanced AI technologies. Techniques including CNNs, Transformers, attention mechanisms, and prompt learning have supported efficient multimodal feature extraction and fusion, while rich multimodal data have in turn facilitated the optimization of data-driven AI models. As an emerging and powerful paradigm in AI, LLMs exhibit unique potential to overcome the bottlenecks of traditional unimodal and multimodal methods. Their strong natural language understanding, cross-modal reasoning capacities, and few-shot learning ability make them particularly suitable for processing complex, incomplete, and heterogeneous clinical data, thereby offering a promising solution to these challenges.

## 5. Detection of Depression Using LLMs

Compared with conventional DL models, LLMs exhibit stronger semantic understanding and contextual reasoning capabilities, which may provide advantages in analyzing patient narratives, emotional expressions, and long-text psychological interactions. These characteristics make LLMs particularly promising for online mental health consultation, intelligent screening systems, and low-resource psychological assessment scenarios. Given these advantages and aiming to address the above limitations of conventional approaches, this section systematically reviews the state of the art in LLM-based depression detection, covering three core research perspectives: the applications of basic LLMs in depression detection, the breakthroughs of optimized LLMs in depression detection, and the innovations of MLLMs in depression detection.

### 5.1. Applications of Basic LLMs in Depression Detection

In the beginning stages of exploration, the primary sources of information employed for identifying depression utilized simple pre-trained language models, among them the depression-related texts with the generalized models with LLMs, such as BERT, GPT-3, and Llama2-7B, without any technical refinements. The primary purpose of this line of research was to assess the potential of LLMs in the extraction of semantic features corresponding to depression and serving simple detection functions.

Xian et al. [88] investigated the LLMs modeling shadow analyses using clinical interview data. They found that DeepSeek-V3 appeared to be the most dependable and most economical model and performed very well in zero-shot and few-shot. In the case of LLMs targeting clinical texts, Lho et al. [89] studied the depression and suicide risk LLMs and text-embedding model effectiveness in SCT narratives in psychotherapy. They studied the efficacy of a number of LLMs (including GPT-4o, Gemini-1.0-pro, and GPT-3.5-turbo-16k) and a number of text-embedding models (e.g., text-embedding-3-large) in SCTs of 1064 patients. In clinical texts, Segin et al. [90] studied perinatal depression (PPD) and expanded studies of PPD chatbots. They investigated the efficacy of LLMs (ChatGPT, Bard) and Google Search against 14 PPD questions governed by the American College of Obstetricians and Gynecologists (ACOG) guidelines. LLMs were found to give clinically correct answers, and people were correct in noting that, in response to ACOG FAQ, ChatGPT was the best.

At this stage, the basic LLMs demonstrate significant shortcomings, such as poor performance in task flexibility in complex scenarios. For example, when looking at complicated clinical situations or social media posts with implicit depressive mentions, LLMs perform poorly in terms of accuracy and stability, and are not specifically optimized for the task of depression detection. Furthermore, the basic LLMs display inadequate depression detection task design optimizations and low performance in specialized tasks, such as quantitative analysis with depression rating scales.

### 5.2. Optimized LLMs for Depression Detection

While LLMs have proven impressive capabilities for detecting depression in varied text and multimodal data, plain, unmodified LLMs still exhibit a lack of sufficient task adaptation, poor interpretability, and underperformance in challenging clinical contexts. To address these limitations, researchers have proposed several targeted optimizations, among which prompt engineering and domain-specific fine-tuning are the most common and effective. In this section, the major advancements pertaining to optimized LLMs and depression detection are analyzed. Focus is placed on the improvements in prompt engineering, fine-tuning, and related performance in real-world scenarios.

#### 5.2.1. Prompt Engineering

Given the limitations of basic LLMs in depression detection, researchers have adopted Prompt Engineering strategies to improve performance. In other words, selective prompt templates or frameworks are employed to help LLMs better understand the task of depression detection and learn relevant features, which can alleviate the limitations of basic models in more complex scenarios and specialized tasks.

In this context, Teferra et al. [91] attempted to answer some of the primary mental health diagnosis problems: the subjective nature of self-report questionnaires, and the inaccurate self-report assessments and self-reports experienced by LLMs. They investigated the possibilities of using zero-shot LLMs for developing depression screening and assessment of the PHQ-8 item scale, using the DAIC-Woz dataset and the RISEN prompt engineering framework, while trying to assess a number of LLMs, including different versions of GPT, Llama3_8B, Cohere, and Gemini. Their research showed the various performance up- or down- sides of the different models, across the dimensions of symptoms. For instance, it was found that the GPT models, especially the GPT-4o, were the best predictive models across the PHQ-8 items, while Llama3_8B was the best predictive model when Anhedonia was the focus, and Cohere was the best predictive model for assessment of psychomotor activity symptoms. This study confirmed the effectiveness of prompt engineering for depression scale assessment and also showed the model-specific focus for optimization of LLMs. In support of this research line, Kim et al. [92] conducted empirical studies on LLMs’ ability to identify depressive and anxiety-related symptoms embedded in safety messages written by diabetic patients. This study used a multi-strategy optimization technique, in which prompting and persona-setting were used to improve the model’s flexibility with clinical text. The authors report that three of the five models demonstrated remarkably high performance, with F1 and accuracy scores over 90%. Most impressively, Llama 3.1 405B achieved 93% in both metrics with zero-shot learning, demonstrating that prompt tuning with supplementary techniques can achieve LLMs’ better performance on symptom detection in clinical text from a particular domain. Wang et al. [93] designed a two-stage LLM-based system with Beck’s Depression Inventory (BDI), a gold standard in depression assessment, after expanding the data source to social media. The authors used text and responses from Reddit to assess depression and ranked and extracted, in the first stage, text responses to depression symptoms from the BDI, and in the second stage, set up a system to auto-assess the depression severity.

#### 5.2.2. Fine-Tuning and Performance Optimization

Beyond prompt engineering, domain fine-tuning has emerged as another pivotal optimization direction for LLMs in depression detection. It adapts LLMs to the specific domain of depression detection by leveraging clinical or depression-related text data, effectively enhancing model adaptability to domain-specific contexts. Domain fine-tuning, which adapts LLMs to the specific task of depression detection by leveraging clinical or depression-related textual data, has emerged as another pivotal optimization direction.

Weber et al. [94] investigate the potential uses of AI and LLMs in mental health studies. In particular, they trained a lightweight German BERT-based LLM with a regression model to predict the individual Montgomery–Askerby Depression Rating Scale (MADRS) scores (0 to 6 in severity) with a regression model. They trained their model on two datasets, one of which included real structured clinical interviews with transdiagnostic patients, and the other was synthetically generated interview transcripts. In their context, Perez-Toro et al. [95] made use of both general-purpose LLMs and mental health applications of LLMs. They investigated the impact of age, gender, and linguistic diversity on the performance of LLM, based on balanced datasets in English, Spanish, and Dutch, in multilingual tasks of depression severity classification. Shao et al. [96] outlined the issues of detection biases as a result of statistical bias and the inability to control the quality of machine-generated corpora, affecting the LLMs trained on synthetic data to screen early mental illnesses such as depression. To alleviate this, they performed the relevant experiments on subtasks using the DepTweet dataset and had three main objectives: to evaluate the reasoning of LLMs, to expose statistical biases, and to seek optimizations using strategies like task cracking, contrastive few-shot learning, chain-of-thought prompt tuning, human annotation, and the filtering of human-preferred detection outcomes. Two significant observations were made as a result of the experiments. To begin with, the accuracy of the LLMs was higher in identifying explicit language related to depression rather than other implicit emotional manifestations, and secondly, the biases of the models were more significant in evaluative judgments of the keywords concerning depression. Lastly, among the two optimization methods considered, supervised fine-tuning (SFT) and direct preference optimization (DPO), it was DPO that resulted in a notable enhancement in the model performance.

Against the backdrop of the high prevalence of depressive and anxiety disorders—conditions that pose severe risks such as elevated suicide rates and socioeconomic burdens if left untreated—and the lingering challenges of high costs and ethical concerns in LLM-based mental health applications, Liu et al. [97] developed a pipeline to synthesize 1157 clinical interview dialogues (PsyInterview) and trained an LLM-driven screening system, EmoScan. This system is capable of distinguishing between coarse-grained and fine-grained emotional disorders while conducting high-quality text-based interviews. To mitigate the subjectivity and resource constraints inherent in current psychiatric diagnosis, Xu et al. [98] explored the use of LLMs to identify symptoms from psychiatrist-patient dialogues (treating these symptoms as intermediate features) for the prediction of diagnostic labels. Based on audio recordings of 1160 outpatients with depression or anxiety, they trained LLMs for symptom identification and scale rating and constructed an ensemble learning pipeline with 10-fold cross-validation to perform diagnosis and symptom classification tasks. Munir Shah et al. [99] investigated fine-tuned LLMs, namely GPT-3.5 Turbo 1106 and LLaMA2-7B, optimizing the application range of LLM-based depression detection from clinical data to social media texts. The first author analyzed social media data and found that the fine-tuned LLMs GPT-3.5 Turbo 1106 and LLaMA2-7B recorded an impressive 96.0% and 84.0% accuracy, respectively, and attributed this to the models being well-optimized, as their performance across the dimensions of recall, precision, and F-score was supported by the data. The fine-tuned LLMs demonstrated reliability and provided an effective framework for the task and confirmed the validity of LLM-based frameworks for early diagnosis of depression based on social media data.

Taken together, prompt engineering and domain fine-tuning have enhanced the capability of LLMs in depression detection, enabling more accurate, reliable, and scenario-adaptive identification of depressive symptoms across clinical interviews, patient-generated texts, and social media data. These optimized strategies not only alleviate the inherent limitations of general LLMs but also provide technical support for early screening, severity assessment, and auxiliary diagnosis of depression. Despite these advances, further improvements in model expressiveness and recognition robustness are still needed. To this end, researchers have increasingly explored MLLMs to advance depression recognition. This section thus focuses on the innovations of MLLMs in this field.

### 5.3. Innovations of MLLMs for Depression Detection

In contrast to simple multimodal input systems, MLLMs take LLMs as the core framework, integrate multimodal information such as text, audio, and vision, and realize the unified understanding and collaborative processing of multimodal information through LLM-driven semantic fusion. Distinct from the multi-feature fusion in the optimization stage, this stage focuses on constructing an LLM-centric deep fusion architecture to achieve the organic integration of multimodal information at the semantic level, rather than mere simple feature concatenation.

#### 5.3.1. Shallow Feature Concatenation

Shallow feature concatenation serves as the basic paradigm of multimodal fusion. It involves independently extracting features from different modalities (text, audio, and vision), followed by concatenation, which are then fed into the model or combined with traditional ML methods.

Existing depression-recognition methods suffer from multiple limitations, including the poor real-world effectiveness of deep neural networks (DNNs), the reliance of LLMs on domain-specific fine-tuning and their incompetence in processing non-textual cues, the inadequacy of text-only detection paradigms, and the lack of integration with psychological expertise. To tackle these extensive limitations, Li et al. [100] presented the pioneering use of LLMs for multimodal depression detection employing the DAIC-Woz dataset. Their method captures audio attributes using the pre-trained Wav2Vec model, aligns these attributes to text-based LLMs for integrated processing, and proposes an innovative method to reduce the previously mentioned shortcomings by infusing psychologic knowledge into LLMs through a tailored Q&A set. Along similar lines, Zhang et al. [101] attempted to tackle the problems of LLMs being text input-centric and the absence of LLMs being used in analyzing depressive states. They proposed a novel framework for multimodal depression detection and incorporated a new modality, called Acoustic Landmarks, which captures speech in the LLM framework. Acoustic Landmarks capture not only speech and enrich text transcripts, but also capture speech micro-expressions and lead to the detection of various emotional states. Their tests on DAIC-Woz proved that the new approach is significantly better than the existing audio-text baseline models, which demonstrates the importance of LLMs and audio features in a novel way. Recognizing the challenges that depression screening tools that rely exclusively on text features present, Jin et al. [102] made another evolution in the multimodal fusion paradigm by incorporating text and audio features. They applied LLMs to design features resulting from text (e.g., indicators of depression severity) and integrated them with scores coming from the Chinese suicide dictionary and emotional audio features obtained by a fine-tuned U-Net. They carried out a comparative study using five ML models, and the best results were obtained with the Random Forest Regression (RFR) model combined with LLM-based Prompt3. There is value to Sadeghi et al. [103], integrating both text and facial attributes. While all fusion approaches are multimodal, many neglect the value added by LLM to text and audio fusion. In multimodal approaches, text and audio fusions increase the efficacy of depression screening. They proposed an innovative, completely automated methodology aimed at ascertaining the depression severity from the E-DAIC dataset. This methodology leveraged LLMs to capture depression-related attributes from interview transcripts and utilized the PHQ-8 scores (intptr) as the supervisory signal to train the depression severity prediction model. They conducted three experimental classifications: text attributes, facial attributes, and the combination of both attributes. For the first time, this study incorporated LLMs to predict the severity of depression and consequently expanded the multimodal research framework.

#### 5.3.2. Deep Semantic Fusion

Deep semantic fusion represents a more advanced research direction. By virtue of LLM-driven cross-modal semantic alignment, it addresses the core issue of “modal feature heterogeneity” inherent in shallow concatenation and has thus evolved into the mainstream paradigm in the field of MLLM research.

To address the lack of transparency regarding how automated depression diagnosis techniques that utilize multimodal interview video data work, and the suboptimal performance of MLLMs due to inadequate training on interview datasets, Zhang et al. [104] introduced MLLM-DR, a novel explainable MLLM comprising a mini-LLM and lightweight query module (LQ-former). In this case, the mini-LLM, which is custom fine-tuned on a robust dataset, produces both depression scores and evaluative reasoning of the scores, and the LQ-former is designed to extract audiovisual features correlated with depression, thereby improving multimodal processing. Results of the experiments indicate that MLLM-DR is the best-performing solution to the CMDC and E-DAIC-Woz benchmark datasets. Tank et al. [105] stated and attempted to mitigate the issues of potential bias and clinician reliance in depression diagnosis using both proprietary and open-source LLMs as well as a new audiovisual multimodal network on the E-DAIC dataset presented in the Audio/Visual Emotion Challenge 2019 [54]. Specifically, the LLMs achieved a remarkable improvement over the current state-of-the-art (SOTA) benchmark, with a root mean square error (RMSE) of 3.98 for text regression tasks and a classification accuracy of 71.43%, while the proposed multimodal network resulted in an RMSE of 6.51 for predicting the PHQ-8 score. Additionally, in order to address the text-centric limitation of the traditional LLMs, which are unable to take in important audiovisual non-verbal cues that are missing and essential for the mental health assessment, and because of the lack of MLLMs designed for psychological purposes, Zhao et al. [106] proposed a new MLLM framework to detect depression. This framework combines an audio language model with visual comprehension and achieves a fine-grained alignment of audiovisual features at the timestamp level, which not only improves the modeling of the cross-modal temporal but also minimizes the data and computational complexities. The DAIC-Woz dataset experiments show that the suggested model improves over single-modal and previous multimodal approaches. Moreover, it enables the inclusion of other physiological signals and suggests more extensive clinical use cases beyond the mental health domain.

MLLMs have brought notable innovations to depression recognition by taking LLMs as the core and integrating text, audio, and visual information through shallow feature concatenation and deep semantic fusion paradigms. These approaches effectively reduce the limitations of traditional unimodal and simple multimodal methods, improve the accuracy and comprehensiveness of depression detection and severity prediction, and provide a promising technical path for intelligent depression assessment.

Overall, LLMs have strongly advanced depression detection research. From basic applications to targeted optimization and multimodal fusion, LLMs have overcome several limitations of traditional methods and delivered more flexible and robust technical solutions for depressive state identification. However, existing work typically assumes complete and standardized multimodal inputs, overlooking the common issue of missing modalities in real-world settings, which can degrade recognition stability and accuracy. To tackle this practical issue, further investigation into incomplete-modal emotion analysis and depression recognition is essential.

## 6. Incomplete Multimodal Learning for Depression Detection: Challenges and Prospects

**Figure 2:**
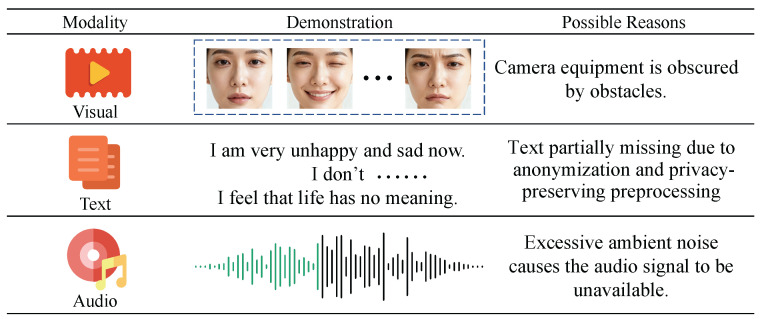
Modal missing example diagram.

Although extensive research has been conducted on depression detection using representative AI technologies including ML, DL, and LLMs, practical deployment often encounters issues such as incomplete facial expression capture, missing segments of emotional speech cues, and interrupted physiological signal acquisition, as illustrated in Figure 2, which may lead to the loss of key information required for depression assessment [107]. Such information loss severely compromises the model’s ability to effectively characterize depressive states, thereby weakening its stability and reliability in real clinical scenarios. To clarify the current research status: despite its practical importance, research specifically addressing missing-modality problems in depression detection remains relatively limited. Accordingly, this section systematically reviews the research progress of incomplete modal emotion analysis and summarizes existing methods for incomplete multimodal depression recognition, followed by discussions on future research directions.

### 6.1. Incomplete Modal Sentiment Analysis

#### 6.1.1. Generative Methods

Generative methods aim to use data from existing modalities to synthesize or reconstruct the data of the missing modalities through generative models, so that their distribution is as similar as possible to the real data. The core of this method is to learn the mapping relationship between modalities. Cai et al. [108] proposed a deep encoder-decoder neural network with auxiliary adversarial loss to address multimodality missing data in clinical applications by formulating the problem as conditional image generation. Zhao et al. [109] proposed a unified solution, namely the Missing-Modality Imagination Network (MMIN), to address the problem of uncertain modality missing; by learning robust joint multimodality representations, this network can predict the representations of any missing modality based on existing modalities under different modality-missing conditions. Zhou et al. [110] proposed an end-to-end deep neural network (with a feature-enhanced generator, correlation constraint block, and multi-encoder U-Net) for brain tumor segmentation under missing MRI modalities. Zhang et al. [111] translated the task of learning multi-view latent representations into a degradation process that mimics data transmission, thereby implicitly achieving an optimal tradeoff between the consistency and complementarity across different views. To handle missing modalities in multimodal data caused by sensor failures, Tran et al. [112] proposed the Cascaded Residual Autoencoder (CRA). This method leverages inter-modal relatedness and the architecture of stacked autoencoders, demonstrating superior performance in both data imputation tasks and subsequent object recognition tasks. Additionally, Wang et al. [113] proposed the IMDer method, which adopts a score-based diffusion model for missing-modality recovery. This effectively mitigates the degradation of the Multimodal Emotion Recognition (MER) performance caused by missing modalities in real-world scenarios.

#### 6.1.2. Joint Learning Methods

Joint Learning methods focus on learning the joint representations of multiple modalities by leveraging the interactions across different modalities.

For example, Han et al. [114] introduced a new model for joint training that is designed to combat the challenges posed by underutilized modalities in monomodal emotion recognition. The model fuses audio and visual data implicitly using a combination of modality networks and a common network, employing auxiliary modality loss and main modality loss.

Beyond foundational fusion frameworks, several methods have advanced toward explicit handling of missing modalities. Luo et al. [115] developed the Multimodal Reconstruct and Align Net (MRAN), which is based on missing index embedding for missing feature reconstruction, the projection of visual/acoustic features to textual space, and the alignment of all features to emotion category word embeddings. In the same spirit, Yuan et al. [116] introduced TFR-Net, a transformer-based architecture that employs intra-, and inter-modal attention extractors to obtain powerful representations of cross-modality and includes a reconstruction module to generate missing features, which improves the flexibility of the architecture for incomplete inputs.

In order to improve robustness to missing modalities, Pham et al. [117] designed the Multimodal Cyclic Translation Network (MCTN) to analyze the sentiment of multiple modalities. Given the fact that all the existing joint representations are susceptible to the noise of missing modalities, the model builds robust joint representations via cyclic translation loss designed in the spirit of Seq2Seq, and thus, sentiment prediction becomes possible with the source modality only at test time and is robust to missing or noisy modalities. For dialogue, specifically, Lian et al. [118] proposed the Graph Complete Network (GCNet) framework, which precisely captures dialogue participant and time dependencies through customized Speaker GNN and Temporal GNN modules. It follows the end-to-end paradigm and performs joint optimization of classification and reconstruction in order to fully leverage complete and incomplete data to improve model quality. Liu et al. [119] proposed a modality translation-based MTMSA model for the uncertain missing modalities in multimodal sentiment analysis (MSA). The model initially translates the visual and audio data to text through a specific modality translation, then integrates the translated modalities with the encoded original text to build Missing Joint Features (MJFs), which ensures the effective fusion despite the incompleteness of the inputs.

### 6.2. Incomplete Modality Depression Detection Methods

Although numerous mature methods for addressing the problem of missing modalities have been developed in the field of sentiment analysis, research on this issue remains scarce in the field of depression detection. Multimodal depression-recognition models generally face the problem of modality incompleteness, which severely limits their robustness. Meanwhile, challenges such as the difficulty in collecting high-quality multimodal data and performance degradation caused by missing/degraded modalities further hinder the clinical application of multimodal depression-recognition technology. To address these challenges, Pan et al. [107] proposed Dis2DR—a novel framework integrating feature disentanglement and privileged knowledge distillation—that disentangles signal features, suppresses noise, transfers knowledge from complete to limited-modal inputs, and achieves superior performance over state-of-the-art methods on four AVEC datasets, including a 9.8% improvement over AVA-DepressNet on AVEC 2013 with single-modality input.

### 6.3. Future Research Prospect

Despite the growing body of research on incomplete multimodal learning and its preliminary applications in depression recognition, this field remains in its infancy and presents several promising directions for future exploration. Despite rapid advances in AI-driven depression detection, several challenges still hinder large-scale clinical adoption, including limited interpretability, insufficient dataset standardization, privacy concerns, and poor robustness under incomplete multimodal conditions. Moreover, two interrelated gaps compound these challenges: the lack of unified data protocols across studies, which undermines model generalizability and fair comparison; and the disconnect between controlled laboratory settings and real-world clinical environments, where patient compliance and data quality issues are prevalent but rarely captured in benchmarks. To bridge these gaps, the following research directions should be prioritized.

First, an adaptive incomplete multimodal fusion framework driven by LLMs should be constructed. By integrating generative models with MLLMs, the adaptive reconstruction of missing physiological and behavioral modalities can be realized, and attention mechanisms can be adopted to dynamically assign weights to available modalities, so as to significantly improve the robustness of the model under various modality-missing patterns.

Second, on this basis, domain-adaptive optimization of LLMs for depression scenarios should be further carried out. Specialized datasets covering diverse modality-missing patterns need to be constructed, and prompt learning strategies integrated with clinical diagnostic criteria should be designed, enabling the model to infer missing information by combining available data and clinical knowledge, thus further enhancing the detection accuracy and interpretability of the model. Yet, the absence of standardized dataset protocols across studies limits model generalizability and cross-method comparability.

Third, to meet the practical demands of clinical and community applications, lightweight MLLMs should be developed. Computational costs can be reduced through model pruning, quantization, and knowledge distillation, so as to achieve real-time depression assessment on edge devices and provide feasible support for the clinical translation of incomplete multimodal detection technology.

Finally, in-depth research should be conducted to improve the clinical reliability of the model. Authoritative psychiatric diagnostic criteria such as PHQ-8, DSM-5, and MADRS should be deeply embedded in the model architecture to guide modality completion and decision-making with professional knowledge, thereby comprehensively enhancing the clinical applicability and diagnostic credibility of the model.

Advancing depression recognition under incomplete multimodal conditions requires a paradigm shift from static fusion models to adaptive, knowledge-aware, and LLM-driven intelligent systems. Addressing these challenges will be key to bridging the gap between research prototypes and practical clinical applications.

### 6.4. Limitations of the Study and Clinical Translation Considerations

Although this review comprehensively summarizes recent advances in depression detection, several limitations should be acknowledged. As a narrative review, the literature selection and synthesis process may inevitably involve a degree of subjectivity, and some recently emerging studies may not have been fully covered due to the rapid evolution of LLM-related research. Furthermore, the existing literature remains largely focused on binary depression identification and severity estimation, with limited attention to heterogeneous depressive subtypes and comorbid psychiatric conditions. In addition, dataset scale and standardization remain insufficient, which limits cross-study comparability and the development of robust benchmarking protocols.

Beyond these methodological limitations, a notable translational gap remains between current LLM-based depression recognition systems and real-world clinical deployment. At present, these systems should be considered to be assistive and screening-oriented tools rather than autonomous diagnostic or therapeutic systems. Accordingly, their role in clinical workflows is better understood as complementary support to clinicians rather than replacements for psychotherapy or pharmacological interventions.

From a clinical translation perspective, formal clinical trials for LLM-assisted depression screening may be considered when models demonstrate consistent cross-domain generalization, robustness under incomplete multimodal conditions, interpretable decision-support behavior, and performance levels that are acceptable for clinical risk management. In such settings, human-in-the-loop evaluation designs involving psychiatrists, psychologists, and other clinical experts are essential to ensure continuous oversight and validation of AI-assisted outputs.

Existing exploratory studies in AI-assisted mental health screening and conversational systems indicate preliminary feasibility; however, large-scale clinically validated deployment remains limited. Potential applications may include early screening, risk stratification, longitudinal emotional monitoring, and supportive assistance within mental healthcare workflows, particularly in regions with limited access to mental health professionals.

Nevertheless, several challenges continue to hinder large-scale clinical adoption. These include privacy protection of sensitive mental health data, potential algorithmic bias, unclear accountability for automated decision-making, and mismatches between screening capacity and available healthcare resources. In addition, large-scale or unsolicited monitoring of personal communications raises important ethical concerns related to informed consent, autonomy, and public trust. These issues highlight the importance of transparent governance and clinician-centered deployment strategies.

Overall, future progress in LLM-assisted depression recognition is expected to depend not only on methodological improvements, but also on rigorous clinical validation, well-defined evaluation frameworks, ethical safeguards, and careful integration into real-world mental healthcare systems.

## 7. Conclusions

This paper provides a comprehensive review of depression detection research, covering its fundamental concepts, clinical evaluation criteria, datasets, and methodological developments. In particular, widely used assessment scales and multimodal datasets are summarized to provide important foundations for subsequent model development and performance evaluation. From a modality-oriented perspective, this review analyzes unimodal and multimodal depression-recognition approaches, highlighting their strengths, limitations, and evolutionary trends. Building upon this foundation, the emerging role of LLMs in depression detection is further discussed, including their applications, optimization strategies, and potential advantages in improving semantic understanding and multimodal fusion. Despite these advances, most existing studies rely on complete multimodal data, which limits real-world clinical robustness. To address this, this review summarizes recent progress in incomplete multimodal learning and prospects future directions for clinically practical depression recognition. However, bridging the gap between laboratory prototypes and clinical implementation remains challenging due to interpretability, data standardization, privacy, and robustness issues. Despite the promising potential of LLM-based depression-recognition systems, their practical deployment still requires rigorous clinical validation, ethical governance, and careful integration with existing mental healthcare workflows. Overall, this work provides a unified perspective on the evolution of depression detection technologies and offers useful references for future research on intelligent mental health assessment.

## Figures and Tables

**Table 1:** Classification Criteria of Common Depression Scales.

Scale	Interview	Self Assessment	Normal	Mild	Moderate	Severe	Very Severe
BDI-II [42]		*√*	0–13	14–19	20–28	29–63	—
PHQ–8 [46]		*√*	0–4	5–9	10–14	15–19	20–24
PHQ–9 [47]		*√*	0–4	5–9	10–14	15–19	20–27
HAMD [48]	*√*		0–7	8–13	14–18	19–22	≥23
QIDs [49]		*√*	0–5	6–10	11–15	16–20	≥21
MADRS [50]	*√*		0–11	12–22	23–30	31–35	≥36
DSM–IV [45]	*√*				11–15	16–20	≥21

*Note:√* indicates the assessment type of each scale. “Interview” refers to clinician-administered assessment, while “Self Assessment” refers to patient self-reported assessment. An em dash (—) means the corresponding severity level is not covered by the scale. The en dash in value ranges (e.g., 0–13) denotes numerical intervals.

**Table 3:** Overview of unimodal and multimodal depression detection paradigms.

Paradigm	Subcategory	Representative Studies
Unimodal	Audio	Arnab Kumar Das et al. [63]; Huang et al. [64]; Wu et al. [65]
	Text	Lyu et al. [66]
	Visual	Fu et al. [67]; Liu et al. [68]; Pan et al. [69]; Song et al. [70]
	EEG	Xi et al. [71]; Ying et al. [72]; Zhang et al. [73]
Multimodal	Physiology & Behavior	Wang et al. [74]; Hamid et al. [75]; Puneet Kumar et al. [76]
	Multi-behavioral Modality	Li et al. [77]; Liu et al. [78]; Iyortsuun et al. [79]; Zhang et al. [80]
	Fusion Strategy Optimization	Zhang et al. [81]; Liu et al. [82]; Yang et al. [83]

## Data Availability

No new data were created or analyzed in this study. Data sharing is not applicable to this article.

## Abbreviations

The following abbreviations are used in this manuscript:

LLM	Large language model
MLLM	Multimodal large language model
ML	Machine learning
AI	Artificial intelligence
DL	Deep learning
CNN	Convolutional Neural Network
